# Crystal structure of 1-ethyl-5-iodo­indolin-2-one

**DOI:** 10.1107/S2056989015009937

**Published:** 2015-05-28

**Authors:** Man Zhang, Yu-Xiang Shen, Qi Fang, Lei Wang, Da-Zhi Li

**Affiliations:** a School of Chemistry and Chemical Engineering, Shandong University, Jinan 250100, People’s Republic of China; b State Key Laboratory of Crystal Materials, Shandong University, Jinan 250100, People’s Republic of China; c Binzhou Key Laboratory of Material Chemistry, Department of Chemical Engineering, Binzhou University, Binzhou 256603, People’s Republic of China

**Keywords:** crystal structure, indolinone derivatives, hydrogen bonding, inter­molecular inter­actions, dipole moment

## Abstract

In the title indolinone derivative, C_10_H_10_INO, all the non-H atoms, except the terminal methyl C atom, are almost coplanar. The mol­ecules are arranged into columns extending along the *a*-axis direction and inter­act with the mol­ecules in adjacent columns *via* C—H⋯O hydrogen bonds [H⋯O distance = 2.57 (3) Å] and I⋯I short contacts of 3.8986 (3) Å. A one-dimensional zigzag iodine chain along the *a* axis is apparent between two neighbouring columns.

## Abstract 2

Mol­ecules of 1-ethyl-5-iodo­indolin-2-one are arranged in columns extending along the *a* axis and inter­act with the mol­ecules in adjacent columns *via* inter­molecular C—H⋯O hydrogen bonds and I⋯I short contacts. A one-dimensional zigzag iodine chain along the *a* axis can be recognized between two neighbouring columns.

## Chemical context   

Indolinone derivatives play an important role in the pharma­ceutical industry and some of them show anti­neoplastic (Cane *et al.*, 2000 ▸), anti­bacterial (Kumar *et al.*, 2013 ▸) and anti-inflammatory (Mammone *et al.*, 2006 ▸) activities. The indolinone skeleton can be also found in many known bioactive drugs, such as horsfiline (Murphy *et al.*, 2005 ▸), rhynchophylline (Deiters *et al.*, 2006 ▸) and the gelsemium alkaloids (Kitajima *et al.*, 2006 ▸). In addition, indolinone derivatives are widely used in the spice industry and agriculture, as functional materials (Ji *et al.*, 2010 ▸) and dye inter­mediates.
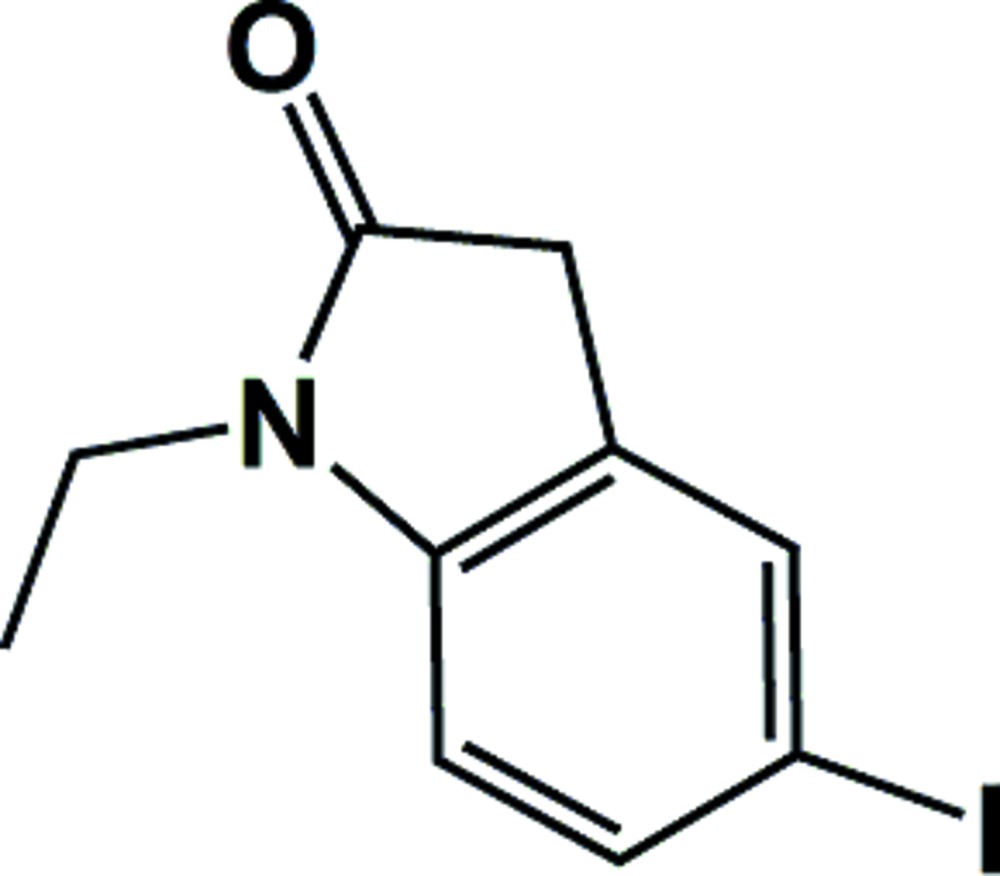


In recent years, the synthesis and crystal structures of many indolinone derivatives have been reported including 6-chloro-5-(2-chloro­eth­yl)oxindole (Nadkarni & Hallissey, 2008 ▸). We have recently synthesized and reported the crystal structures of several indolin-2-one derivatives including 1-phenyl-indolin-2-one (Wang *et al.*, 2015 ▸). As a continuation of our work in this field, we report here the synthesis and crystal structure of the title compound, 1-ethyl-5-iodo­indolin-2-one.

## Structural commentary   

**Figure 1:**
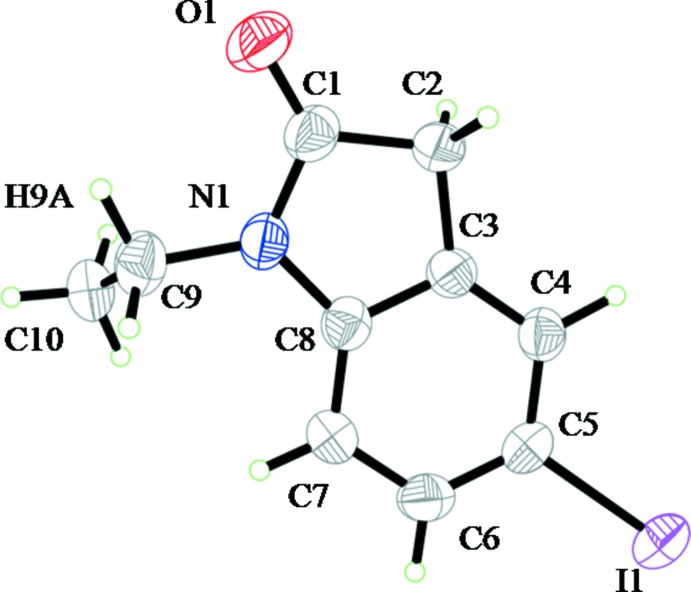
The mol­ecular structure of the title compound. Displacement ellipsoids are drawn at the 50% probability level.

The title mol­ecule is shown in Fig. 1 ▸. The non-H atoms of the indoline core are virtually coplanar [mean deviation is 0.011 (3) Å with a maximum deviation of 0.023 (3) Å for C1]. The atoms C9, O1 and I1 are essentially co-planar with the indoline core, with deviations of 0.019 (4) Å for C9, 0.070 (3) Å for O1, and 0.127 (1) Å for I1. The sum of valence angles around N1 is 360.0°, indicating an *sp* ^2^ hybridization of this atom. The two C—N bonds in the five-membered ring have a partial double-bond character [N1 C1 1.370 (4) Å; N1 C8 1.400 (3) Å], indicating conjugation of the π-electrons of the NC=O group with the π-electrons of the benzene ring.

## Supra­molecular features   

**Figure 2:**
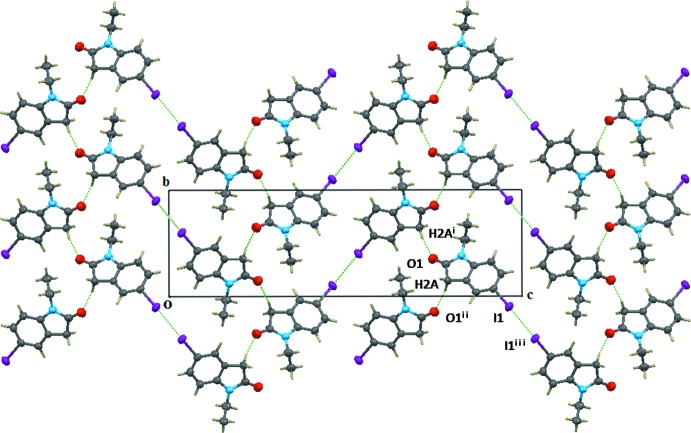
The view of the structure along the *a* axis, showing the C—H⋯O hydrogen bond between columns and the I⋯I inter­actions between columns. [Symmetry codes: (i) −*x* + 3, *y* + 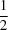, −*z* + 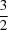; (ii) −*x* + 3, *y* − 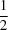, −*z* + 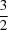; (iii) *x* − 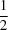, −*y* − 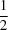, −*z* + 2.]

**Figure 3:**
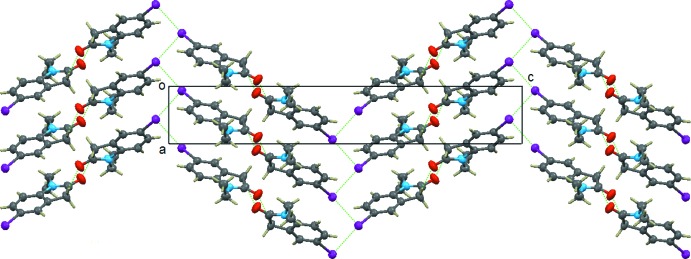
The view of the structure along the *b* axis, showing the one-dimensional columnar structure and the zigzag iodine chains along the *a* axis.

The crystal packing in the title compound is shown in Figs. 2 ▸ and 3 ▸. The mol­ecules are face-to-face parallel-packed forming a column along the *a* axis with π–π inter­actions centroid–centroid distances = 4.130 (2) and 4.462 (2) Å]. Mol­ecules from neighbouring columns are connected by a C—H⋯O hydrogen bond (Table 1 ▸) with the formation of a layer-type aggregate parallel to (001). There is an I⋯I contact shorter than the sum (3.96 Å) of the van der Waals radii [I⋯I^i^ 3.8986 (3) Å, C—I⋯I^i^ 173.3 (3)°; symmetry code: (i) *x* − 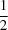, −*y* − 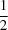, −*z* + 2] joining the columns of mol­ecules in adjacent layers and forming a kind of 1-D zigzag chain along the *a*-axis direction (see Fig. 3 ▸). An important feature of the columns is that they are polar, *i.e*. all mol­ecular dipole moments in the same column point in the same direction.

DFT/b3lyp/genecp calculations were carried out, which took the pseudopotential basis set LanL2DZ for the iodine atom and the 6–311g(d) basis set for the other atoms, to optimize the mol­ecular geometry and calculate the dipole moment using the *GAUSSIAN03* program (Frisch *et al.*, 2003 ▸). The dipole moment of the title mol­ecule (1.707 D) is much smaller than that of its precursor mol­ecule, 1-ethyl-5-iodo­indolin-2,3-dione (5.432 D). This difference may partly explain the non-centrosymmetry of the title crystal (space group *P*2_1_2_1_2_1_) and the centrosymmetry of the crystal of the precursor (Wang *et al.*,2014 ▸). On the other hand, the non-centrosymmetry of the title crystal may be better explained by the I⋯I inter­molecular inter­actions, for there are no I⋯I short contacts in the above centrosymmetric precursor crystal.

## Database survey   

A search of the Cambridge Structural Database (WebCSD, Version 5.36; last update April 2015; Groom & Allen, 2014 ▸) for 5-iodo­indolin-2-one derivatives gave 15 hits. Of these 16 structures (with the title structure included), the number of non-centrosymmetric structures (9) is slightly greater than the number of centrosymmetric structures (7). In these 16 structures, there are four structures which exhibit I⋯I short inter­molecular contacts and all the four structures are non-centrosymmetric (three of them belong to the *P*2_1_2_1_2_1_ space group and the other one belongs to the *P*6_3_ space group; Takahashi *et al.*, 2014 ▸). Therefore, the I⋯I contacts seem to promote non-centrosymmetric packing in this kind of compound.

## Synthesis and crystallization   

The title compound was synthesized by reduction of the precursor with an 80% hydrazine hydrate (see reaction scheme) . 1-Ethyl-5-iodo­indolin-2,3-dione precursor (1.714 g, 5.69 mmol) and 80% NH_2_NH_2_·H_2_O (19.0 mL) were added into a 50 mL flask and the mixture was stirred under reflux. The reaction progress was tracked by TLC. After 4.5 h, the reaction mixture was cooled down and poured into 100 mL water with precipitation of yellow solid. Then the mixture was extracted with CH_2_Cl_2_, the organic phase was washed with water and dried with MgSO_4_. The solvent was removed under reduced pressure and the crude product was purified by silica gel column chromatography with CHCl_3_ as eluent. The title compound was obtained as a colorless solid (1.509 g, yield 92.3%). m.p. 403–404 K. Crystals suitable for X-ray diffraction were obtained by slow evaporation of a CHCl_3_ solution.
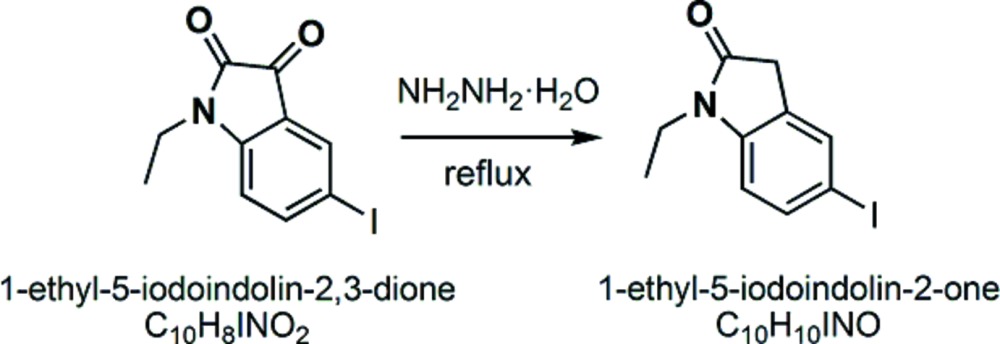


## Refinement   

Crystal data, data collection and structure refinement details are summarized in Table 2 ▸. H atoms bound to aromatic C atoms and methyl­ene C atoms were located in difference maps and freely refined, leading to C—H distances of 0.91 to 1.02 Å. The three H atoms bound to methyl C atoms could also be located in difference maps but they were placed at calculated positions and treated using a riding-model approximation with C—H = 0.96 Å and *U*_iso_(H) = 1.5 *U*_eq_(C).

## Supplementary Material

Crystal structure: contains datablock(s) I. DOI: 10.1107/S2056989015009937/gk2630sup1.cif

Structure factors: contains datablock(s) I. DOI: 10.1107/S2056989015009937/gk2630Isup2.hkl

Supporting information file. DOI: 10.1107/S2056989015009937/gk2630Isup3.cml

CCDC reference: 1028641

Additional supporting information:  crystallographic information; 3D view; checkCIF report

## Figures and Tables

**Table 1:** Hydrogen-bond geometry (, )

*D*H*A*	*D*H	H*A*	*D* *A*	*D*H*A*
C2H2*A*O1^i^	0.99(3)	2.57(4)	3.554(4)	169(3)

Symmetry code: (i) 
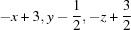
.

**Table 2:** Experimental details

Crystal data	
Chemical formula	C_10_H_10_INO
*M*_r_	287.09
Crystal system, space group	Orthorhombic, *P*2_1_2_1_2_1_
Temperature (K)	295
*a*, *b*, *c* ()	4.4622(1), 8.2664(2), 27.4400(5)
*V* (^3^)	1012.16(4)
*Z*	4
Radiation type	Mo *K*
(mm^1^)	3.12
Crystal size (mm)	0.42 0.32 0.16

Data collection	
Diffractometer	Bruker APEXII CCD
Absorption correction	Multi-scan (*SADABS*; Bruker, 2005 ▸)
*T*_min_, *T*_max_	0.354, 0.635
No. of measured, independent and observed [*I* > 2(*I*)] reflections	12175, 2938, 2878
*R*_int_	0.020
(sin /)_max_ (^1^)	0.704

Refinement	
*R*[*F* ^2^ > 2(*F* ^2^)], *wR*(*F* ^2^), *S*	0.021, 0.050, 1.21
No. of reflections	2938
No. of parameters	148
H-atom treatment	H atoms treated by a mixture of independent and constrained refinement
_max_, _min_ (e ^3^)	0.47, 0.69
Absolute structure	Flack (1983 ▸), 1183 Friedel pairs
Absolute structure parameter	0.02(2)

Computer programs: *APEX2* and *SAINT* (Bruker, 2005 ▸), *SHELXS97*, *SHELXL97* and *SHELXTL* (Sheldrick, 2008 ▸).

## supplementary crystallographic information

### Crystal data

**Table:**

C_10_H_10_INO	*D*_x_ = 1.884 Mg m^−^^3^
*M**_r_* = 287.09	Melting point = 403–404 K
Orthorhombic, *P*2_1_2_1_2_1_	Mo *K*α radiation, λ = 0.71073 Å
Hall symbol: P 2ac 2ab	Cell parameters from 9948 reflections
*a* = 4.4622 (1) Å	θ = 2.5–30.0°
*b* = 8.2664 (2) Å	µ = 3.12 mm^−^^1^
*c* = 27.4400 (5) Å	*T* = 295 K
*V* = 1012.16 (4) Å^3^	Parallelepiped, orange
*Z* = 4	0.42 × 0.32 × 0.16 mm
*F*(000) = 552	

### Data collection

**Table:**

Bruker APEXII CCD diffractometer	2938 independent reflections
Radiation source: fine-focus sealed tube	2878 reflections with *I* > 2σ(*I*)
Graphite monochromator	*R*_int_ = 0.020
Detector resolution: 8.3 pixels mm^-1^	θ_max_ = 30.0°, θ_min_ = 2.6°
ω scans	*h* = −6→6
Absorption correction: multi-scan (*SADABS*; Bruker, 2005)	*k* = −11→9
*T*_min_ = 0.354, *T*_max_ = 0.635	*l* = −37→33
12175 measured reflections	

### Refinement

**Table:**

Refinement on *F*^2^	Hydrogen site location: mixed
Least-squares matrix: full	H atoms treated by a mixture of independent and constrained refinement
*R*[*F*^2^ > 2σ(*F*^2^)] = 0.021	*w* = 1/[σ^2^(*F*_o_^2^) + (0.0144*P*)^2^ + 0.4592*P*] where *P* = (*F*_o_^2^ + 2*F*_c_^2^)/3
*wR*(*F*^2^) = 0.050	(Δ/σ)_max_ = 0.002
*S* = 1.21	Δρ_max_ = 0.47 e Å^−^^3^
2938 reflections	Δρ_min_ = −0.69 e Å^−^^3^
148 parameters	Extinction correction: *SHELXL97* (Sheldrick, 2008), Fc^*^=kFc[1+0.001xFc^2^λ^3^/sin(2θ)]^-1/4^
0 restraints	Extinction coefficient: 0.0014 (3)
Primary atom site location: structure-invariant direct methods	Absolute structure: Flack (1983), 1183 Friedel pairs
Secondary atom site location: difference Fourier map	Absolute structure parameter: 0.02 (2)

### Special details

**Table:**

Experimental. Scan width 0.4° ω, Crystal to detector distance 6.20 cm, exposure time 20 s, 17 h for data collection
Geometry. All e.s.d.'s (except the e.s.d. in the dihedral angle between two l.s. planes) are estimated using the full covariance matrix. The cell e.s.d.'s are taken into account individually in the estimation of e.s.d.'s in distances, angles and torsion angles; correlations between e.s.d.'s in cell parameters are only used when they are defined by crystal symmetry. An approximate (isotropic) treatment of cell e.s.d.'s is used for estimating e.s.d.'s involving l.s. planes.
Refinement. Refinement of *F*^2^ against ALL reflections. The weighted *R*-factor *wR* and goodness of fit *S* are based on *F*^2^, conventional *R*-factors *R* are based on *F*, with *F* set to zero for negative *F*^2^. The threshold expression of *F*^2^ > σ(*F*^2^) is used only for calculating *R*-factors(gt) *etc*. and is not relevant to the choice of reflections for refinement. *R*-factors based on *F*^2^ are statistically about twice as large as those based on *F*, and *R*- factors based on ALL data will be even larger.

### Fractional atomic coordinates and isotropic or equivalent isotropic displacement parameters (Å^2^)

**Table:**

	*x*	*y*	*z*	*U*_iso_*/*U*_eq_	
I1	0.57349 (4)	−0.10015 (2)	0.963176 (7)	0.05126 (7)	
C5	0.7961 (6)	0.0703 (3)	0.91955 (9)	0.0404 (5)	
O1	1.3877 (6)	0.3521 (3)	0.74792 (8)	0.0644 (7)	
C4	0.8354 (6)	0.0383 (3)	0.87003 (10)	0.0410 (5)	
C3	0.9948 (5)	0.1476 (3)	0.84294 (9)	0.0386 (6)	
C8	1.1096 (6)	0.2881 (3)	0.86420 (9)	0.0394 (5)	
N1	1.2652 (5)	0.3781 (3)	0.82916 (8)	0.0429 (5)	
C9	1.4198 (8)	0.5309 (4)	0.83826 (12)	0.0505 (6)	
C10	1.2102 (8)	0.6717 (4)	0.84543 (12)	0.0539 (7)	
H10A	1.0772	0.6795	0.8180	0.081*	
H10B	1.3242	0.7698	0.8482	0.081*	
H10C	1.0955	0.6557	0.8746	0.081*	
C1	1.2634 (7)	0.3027 (3)	0.78470 (10)	0.0472 (6)	
C2	1.0825 (9)	0.1476 (3)	0.78985 (10)	0.0488 (6)	
C7	1.0700 (8)	0.3218 (3)	0.91288 (10)	0.0456 (5)	
C6	0.9096 (8)	0.2096 (4)	0.94064 (9)	0.0472 (6)	
H2A	1.216 (8)	0.054 (4)	0.7827 (11)	0.053 (9)*	
H2B	0.899 (12)	0.165 (5)	0.7685 (17)	0.103 (16)*	
H4	0.754 (8)	−0.062 (4)	0.8534 (12)	0.058 (9)*	
H6	0.884 (8)	0.234 (4)	0.9737 (11)	0.051 (9)*	
H7	1.143 (7)	0.419 (4)	0.9266 (11)	0.053 (9)*	
H9A	1.548 (9)	0.555 (4)	0.8089 (13)	0.067 (11)*	
H9B	1.528 (10)	0.520 (5)	0.8662 (14)	0.075 (12)*	

### Atomic displacement parameters (Å^2^)

**Table:**

	*U*^11^	*U*^22^	*U*^33^	*U*^12^	*U*^13^	*U*^23^
I1	0.05236 (10)	0.05416 (11)	0.04726 (10)	−0.00328 (9)	0.00305 (9)	0.01516 (8)
C5	0.0395 (12)	0.0407 (14)	0.0410 (12)	0.0021 (10)	0.0017 (10)	0.0086 (10)
O1	0.0785 (16)	0.0576 (13)	0.0570 (13)	−0.0032 (13)	0.0277 (13)	0.0062 (10)
C4	0.0445 (13)	0.0333 (12)	0.0451 (13)	0.0004 (9)	0.0016 (10)	0.0007 (10)
C3	0.0434 (14)	0.0350 (12)	0.0374 (12)	0.0044 (8)	0.0043 (9)	−0.0006 (9)
C8	0.0386 (12)	0.0356 (11)	0.0440 (12)	0.0026 (10)	0.0029 (10)	0.0009 (10)
N1	0.0479 (11)	0.0335 (11)	0.0473 (11)	−0.0035 (10)	0.0100 (10)	−0.0003 (9)
C9	0.0432 (13)	0.0488 (15)	0.0596 (17)	−0.0113 (14)	0.0002 (15)	0.0038 (12)
C10	0.0615 (18)	0.0403 (15)	0.0598 (17)	−0.0119 (14)	−0.0008 (15)	−0.0031 (13)
C1	0.0537 (16)	0.0393 (13)	0.0486 (15)	0.0047 (13)	0.0125 (13)	0.0039 (12)
C2	0.0664 (17)	0.0389 (13)	0.0412 (13)	−0.0003 (15)	0.0159 (15)	−0.0040 (10)
C7	0.0526 (14)	0.0414 (13)	0.0428 (13)	−0.0052 (14)	−0.0023 (13)	−0.0048 (10)
C6	0.0530 (14)	0.0538 (15)	0.0349 (12)	−0.0018 (15)	0.0015 (13)	0.0009 (11)

### Geometric parameters (Å, º)

**Table:**

I1—C5	2.099 (3)	C9—C10	1.506 (5)
C5—C6	1.384 (4)	C9—H9A	1.01 (4)
C5—C4	1.395 (4)	C9—H9B	0.91 (4)
O1—C1	1.222 (3)	C10—H10A	0.9600
C4—C3	1.369 (4)	C10—H10B	0.9600
C4—H4	1.02 (3)	C10—H10C	0.9600
C3—C8	1.397 (4)	C1—C2	1.521 (4)
C3—C2	1.508 (4)	C2—H2A	0.99 (3)
C8—C7	1.376 (4)	C2—H2B	1.02 (5)
C8—N1	1.400 (3)	C7—C6	1.398 (4)
N1—C1	1.370 (4)	C7—H7	0.95 (3)
N1—C9	1.461 (4)	C6—H6	0.94 (3)
			
C6—C5—C4	121.3 (2)	C9—C10—H10A	109.5
C6—C5—I1	119.53 (19)	C9—C10—H10B	109.5
C4—C5—I1	119.2 (2)	H10A—C10—H10B	109.5
C3—C4—C5	118.0 (3)	C9—C10—H10C	109.5
C3—C4—H4	118.8 (19)	H10A—C10—H10C	109.5
C5—C4—H4	123.3 (19)	H10B—C10—H10C	109.5
C4—C3—C8	120.8 (2)	O1—C1—N1	125.5 (3)
C4—C3—C2	131.2 (3)	O1—C1—C2	126.8 (3)
C8—C3—C2	108.0 (2)	N1—C1—C2	107.7 (2)
C7—C8—C3	121.8 (3)	C3—C2—C1	103.1 (2)
C7—C8—N1	128.5 (3)	C3—C2—H2A	110.2 (18)
C3—C8—N1	109.7 (2)	C1—C2—H2A	108 (2)
C1—N1—C8	111.5 (2)	C3—C2—H2B	110 (3)
C1—N1—C9	123.3 (2)	C1—C2—H2B	105 (3)
C8—N1—C9	125.2 (2)	H2A—C2—H2B	118 (3)
N1—C9—C10	113.4 (3)	C8—C7—C6	117.4 (3)
N1—C9—H9A	107 (2)	C8—C7—H7	121 (2)
C10—C9—H9A	108 (2)	C6—C7—H7	121.6 (19)
N1—C9—H9B	108 (3)	C5—C6—C7	120.8 (2)
C10—C9—H9B	107 (3)	C5—C6—H6	122 (2)
H9A—C9—H9B	113 (3)	C7—C6—H6	117 (2)
			
C6—C5—C4—C3	1.2 (4)	C8—N1—C1—O1	−178.2 (3)
I1—C5—C4—C3	−176.70 (19)	C9—N1—C1—O1	0.1 (5)
C5—C4—C3—C8	−1.1 (4)	C8—N1—C1—C2	1.3 (3)
C5—C4—C3—C2	178.5 (3)	C9—N1—C1—C2	179.6 (3)
C4—C3—C8—C7	0.5 (4)	C4—C3—C2—C1	−178.6 (3)
C2—C3—C8—C7	−179.1 (3)	C8—C3—C2—C1	1.0 (3)
C4—C3—C8—N1	179.4 (2)	O1—C1—C2—C3	178.1 (3)
C2—C3—C8—N1	−0.3 (3)	N1—C1—C2—C3	−1.4 (3)
C7—C8—N1—C1	178.0 (3)	C3—C8—C7—C6	0.0 (4)
C3—C8—N1—C1	−0.7 (3)	N1—C8—C7—C6	−178.6 (3)
C7—C8—N1—C9	−0.2 (5)	C4—C5—C6—C7	−0.7 (5)
C3—C8—N1—C9	−178.9 (3)	I1—C5—C6—C7	177.2 (2)
C1—N1—C9—C10	108.7 (3)	C8—C7—C6—C5	0.0 (5)
C8—N1—C9—C10	−73.2 (4)		

### Hydrogen-bond geometry (Å, º)

**Table:**

*D*—H···*A*	*D*—H	H···*A*	*D*···*A*	*D*—H···*A*
C2—H2*A*···O1^i^	0.99 (3)	2.57 (4)	3.554 (4)	169 (3)

Symmetry code: (i) −*x*+3, *y*−1/2, −*z*+3/2.
